# The Effect of Urinary Incontinence on Quality of Life of Women at Childbearing Age in Jeddah, Saudi Arabia

**DOI:** 10.5539/gjhs.v8n2p281

**Published:** 2015-07-20

**Authors:** Marwan A. Bakarman, Sadiah Saeed Al-Ghamdi

**Affiliations:** 1 Rabigh Medical College, King Abdulaziz University, Jeddah, KSA; 2 Minisrty of Health, Primary Health Care, Jeddah, KSA

**Keywords:** urine incontinence, quality of life, Saudi women, childbearing age

## Abstract

To estimate the prevalence of urinary incontinence among women of childbearing age at Maternity and Children’s Hospital (MCH), Jeddah, 2012, and to assess its impact on the quality of their life. A cross– sectional analytic approach was carried out among women of childbearing age seen at MCH, Jeddah. Systematic random sampling technique was followed. Each woman fulfilling the inclusion criteria was invited to enroled in the study, the inclusion criteria were women aged 15-50 years, agreed to participate in the study, the exclusion criteria were pregnant women and patients who are seriously ill. Self-administered questionnaire using the King’s Health Questionnaire was utilized to measure Health Related Quality of Life (HRQOL) of patients with UI. Out of 1200 patients attending the gynecology clinic in the MCH, 412 (34.3%) were diagnosed as having UI. Their age ranged between 15 and 50 years with a mean of 34.3 + 7.2 years. Almost 50% indicated that UI affected them badly as wife, mother, their emotions, and their physical and social activities. The most commonly occurring problems were frequent micturition (88.3%), nocturnal enuresis (87.9%). The least occurring, were kidney problems (38.6%) and dripping during sexual activities (40.8%). Increasing age and higher parity were significantly associated with limitations in different life domains. Urinary incontinence is common and often disturbing for Saudi women. It adversely impaired their quality of life.

## 1. Introduction

The definition of urinary incontinence (UI) is the complaint of involuntary loss of urine (Hylen, 2010). Urinary incontinence can be classified as: Stress incontinence which is the involuntary loss of urine on exertion or effort, sneezing, or coughing, Urgency incontinence is the involuntary loss of urine accompanied by or immediately preceded by urgency (Hylen, 2010). Mixed incontinence is the involuntary loss of urine associated with urgency and with effort, exertion, coughing or sneezing (Faltin, 2009). Urinary incontinence was experienced by more than 30% of adult women, and Stress urinary incontinence alone accounts for up to half of all cases; generally the prevalence of UI considered to be from 20% to 50% with the peak to be in the childbearing age group (up to 40%) and then the prevalence increasing in elderly to reach to 50%. In Saudi Arabia a local study done in 2008 at a primary care center in Jeddah found that the prevalence of UI was 41.4 % (Brasha, 2008). This local research studied the prevalence of UI, the risk factors and the barriers to seek health advices but not relating UI to quality of life.

Urinary incontenance remains a silent problem as a significant number of women do not seek treatment, even when their symptoms cause major distress and hinder their daily activities (Riss & Kargl, 2011). While incontinence does not lead to death, it can have a profound effect on quality of life comparable to that of stroke, arthritis and chronic-obstructive pulmonary disease. In addition, incontinence accounts for more than $20 billion in annual expenditures in the United States, an amount greater than the annual direct cost of breast, ovarian, cervical, and uterine cancers (Barber et al., 2005).

One of the major risk factors for stress incontinence is vaginal child birth, it was reported that one third of female experienced stress incontinence 5 years after their first vaginal delivery (Riss & Kargl, 2011).

The term Quality of life is used generally to indicate ‘happiness’, but for every patient it may have different meaning: high income and money, good family life and relationship with others, job satisfaction, good physical and mental health (Falten, 2008).

Urinary incontinence can affect the patient’s life in many ways, some women miss the ability to practice their favorite sports, some are bothered by having to wear sanitary protection, and others their sex life and intimate relationship are much affected by the negative impact of urinary incontinence (Senra, 2015; Babic, 2015).

Although it is still important to do researches in the diagnostic procedure of urinary incontinence, nowadays the focus of new researches has been shifted from only describing and evaluating the phenomenon of urinary incontinence to ask more details regarding the different kinds of urinary incontinence and how it affects the quality of life of a woman (Frick et al., 2009). Therefore our objectives in this study were to estimate the prevalence and assess the impact of different clinical types of incontinence on quality of life among women of childbearing age at Maternity and Children’s Hospital, Jeddah, Saudi Arabia, 2012. We expected that exploring quality of life of those women helps us to better understand what our patients feel and what their real needs and to draw the attention of family physicians to the importance of identifying the population at risk in an attempt for early diagnosis and proper intervention

## 2. Methodology

### 2.1 Research Desigin and Subjects

This cross-sectional analytic study done in the Maternity and Children’s Hospital (MCH), which is the biggest hospital in Jeddah City in Saudi Arabia (SA). There are approximately 44,703 patients attending the Gynecology clinics and about 6,695 patients for Obstetric clinics yearly.

The total monthly number of patients attending the Gynecology clinics was estimated to be roughly ± 4,000 patients, and we selected 30% of this number as a sample size for our study which was equal to 1,200 patients.

### 2.2 Sampling

Systematic random sampling technique was followed (every third patient was selected). Each assigned woman aged 15-50 years old, Saudi and non-Saudi and agree to participate in the study was invited to be enrolled in the study after describing the aim of the study for her. The exclusion criteria were pregnant women and patients who are seriously ill. Those who agreed were requested to fill the Self-administered questionnaire.

### 2.3 Reseacrch Insturment

Those who agreed were requested to fill the Self-administered questionnaire. The Kings Health Questionnaire is a disease specific health related quality of life (HRQOL) instrument to measure quality of life in patients with urinary incontinence (El-Azab, Mohammed & Sabra, 2007). It is used in many clinical studies and was proven to be valid and reliable (Brasha, 2008). It was translated into Arabic then again translated into English to ensure lexical equivalence.

### 2.4 Data Analysis

Data entry and statistical analyses were done using statistical software package SPSS 16.0.

## 3. Results

Out of the 1,200 patients, there were 412 (34.3%) diagnosed as having urinary incontinence. Table (1) demonstrates that 14.6% of the patients indicated that they are complaining of chronic diseases e.g. diabetes, hypertension and bronchial asthma. 10.9% of the patients pointed out that they had previous surgical operations in the pelvis not including caesarean sections (CSs). Meanwhile, it was found that 105(25.5%) of the patients had previous sections, out of them there were 13.6% who had two or more CSs. Only 22(5.3%) of the patients were current smokers.

**Table 1:** Relevant medical history of the patients (n=412)

	No.	%
***Chronic diseases***		
Yes	60	14.6
No	352	85.4
***Previous surgical operation in the pelvis***		
Yes	45	10.9
No	367	89.1
***Previous caesarean section***		
No	307	74.5
Yes	105	25.5
One caesarean section	49	11.9
2-3 caesarean section	35	8.5
4+ caesarean sections	21	5.1
***Smoking status***		
Yes	22	5.3
No	390	94.7

Table (2) demonstrates that, in general, almost one half of the patients indicated that urine incontinence affects badly their role, physical and social activities and their emotions. The effect was remarkable on job and daily outdoor activities, where 20.2% of the patients expressed that they were moderately or a lot affected. This was followed by the effect on ability to travel where the equivalent percentage accounted for 16.5%. Considering the effect on emotions, it was realized that 19.7% of the patients confessed that they are moderately or a lot of time feeling anxious or nervous.

**Table 2:** Effect of urine incontinence on different aspects of life and emotions of the patients

	Response							

	Not at all		Slightly		Moderately		A lot		

	No.	%	No.	%	No.	%	No.	%
***Role limitations***								
Affect household task	217	52.7	152	36.9	37	9.0	6	1.5
Affec job, or normal daily outdoor activities	222	53.9	107	26.0	70	17.0	13	3.2
***Physical and social limitations***								
Affect physical activities	201	48.8	153	37.1	54	13.1	4	1.0
Affect ability to travel	231	56.1	113	27.4	59	14.3	9	2.2
Limit social life	228	55.3	127	30.8	50	12.1	7	1.7
Limit ability to contact friends	236	57.3	112	27.2	55	13.3	9	2.2
***Emotion***								
Feeling depressed	215	52.2	147	35.7	45	10.9	5	1.2
Feeling anxious or nervous	219	53.2	112	27.2	70	17.0	11	2.7
Feeling bad about self	252	61.2	107	26.0	40	9.7	13	3.2
***Sleep and energy***								
Affect sleep pattern	137	33.3	224	54.4	43	10.4	8	1.9
Feeling tired and exhausted	155	37.6	191	46.4	53	12.9	13	3.2
***Personal relationship***								
Affect relation with partner (n=216)	117	54.2	76	35.2	21	9.7	2	0.9
Affect sex life (n=278)	131	47.1	84	30.2	52	18.7	11	4.0
Affect family life (n=216)	124	57.4	63	29.2	22	10.2	7	3.2

On the other hand, a higher proportion of the patients (almost two thirds) reported that urine incontinence affects their sleeping and energy; 12.3% patients pointed out that they were moderately or a lot of time having their sleep pattern affected and 16.1% were feeling tired and exhausted. Regarding effect of incontinence on personal relationships, only patients with whom the items were applicable, are displayed. It was noticed that sexual life was the most affected; where 52.9% of the patients indicated that they suffered in various degrees, out of them 18.7% reported moderate effect and 4% described the effect as being “a lot”.

Table (3) illustrates that the most commonly occurring problems among the study group were frequently going to the WC (88.3%) followed by getting up at night to pass urine and dripping associated with physical exercises (87.9%); and the least to occur were the kidney problems (38.6%) and dripping on sexual activities (40.8%).

**Table 3:** Problems resulting from urine incontinence

Problems	Response					

	Little		Moderate		A lot	

	No.	%	No.	%	No.	%
Frequent going to WC (n=364; 88.3%)	167	45.9	182	50.0	15	4.1
Getting up at night to pass urine (n=362; 87.9%)	176	48.6	158	43.6	28	7.7
Difficulty in controlling desire to urinate (n=319; 77.4%)	139	43.6	165	51.7	15	4.7
Dripping accompanied by strong desire to urinate (n=302; 73.3%)	149	49.3	139	46.0	14	4.6
Dripping associated with physical exercise (n=362; 87.9%)	157	43.4	178	49.2	27	7.5
Nocturnal Enuresis (n=224; 54.4%)	150	67.0	65	29.0	9	4.0
Dripping on sexual activities (n=168; 40.8%)	94	56.0	43	25.6	31	18.5
Kidney problem (n=159; 38.6%)	148	93.1	9	5.7	2	1.3
Pain in urinary bladder (n=208; 50.5%)	181	43.9	192	46.6	28	6.8

Table (4) describes the degree of limitations in different life domains calculated by summating sub items representing collectively each domain and presented in percentages. It was evident that the most affected domain was sleep and energy where the percentage of limitations reached 27.1% and the least affected was the social domain where the limitation accounted for 17.5%.

**Table 4:** The mean percentages of limitations in different life domains resulting from urine incontinence

Different life domains	Mean percentage	SD
Role limitations	21.4	25.1
Physical limitations	21.5	23.9
Social limitations	17.5	22.9
Personal relationships	19.1	14.0
Emotions	20.6	24.1
Sleep and energy	27.1	22.8
Severity measures	23.1	19.4

From table (5), it is obvious that the percentages of limitations were higher among patients who reported that they are suffering from chronic diseases other than incontinence in all life domains. However, these differences were only statistically significant for the percentages of physical limitations p<0.05.

**Table 5:** Differences in the mean percentages of limitations in different life domains according to concurrent chronic diseases, rather than incontinence among the patients

Different life domains’ limitations	Concurrent chronic diseases		P*

	Yes	No	
Role limitation	27.2+26.6	20.5+24.7	0.053
Physical limitation	27.5+25.8	20.5+23.5	0.035
Social limitation	19.4+26.6	17.2+22.2	0.480
Personal limitation	21.1+25.3	18.7+23.8	0.552
Emotions	20.0+20.8	20.7+24.6	0.882
Sleep and energy	26.1+23.2	27.3+22.7	0.715
Severity measure	26.3+21.1	22.6+19.0	0.178

* Based on independent sample t test.

## 4. Discussion

Urinary incontinence is not a life threatening disease, but usually accompanied by a negative impact on quality of life of the patient. UI has a physical and emotional affect, while at the same time it creates an additional financial burden.

Urinary Incontinence can seriously affect the social and personal life of patients, and ultimately their quality of life (Ghafouri, 2014; Minassian, 2015). Women with urine incontinence avoid social gatherings, as they are afraid that sudden loss of urine will put them in an embarrassing situation (Son, 2012).

In the present study, overall, there was a limitation in all domains of quality of life. The most affected domain was sleep and energy where the percentage of limitations reached 27.1% and the least affected was the social domain where the limitation accounted for 17.5%. In Hong Kong Study (Pang 2005) impaired QOL, as assessed by IIQ-7, was reported at 11.7%, while in Iran women with mixed incontinence reported significantly lower QOL and mental health (P < 0.001) compared to those with stress and urge incontinence (Mallah, 2014).

We observed that almost half of the affected women found UI disturbing and claimed it had a detrimental effect on their daily functioning (household tasks and outdoor activities). In other studies, up to 60% of affected women were disturbed or worried by UI (Lopez, 2009; Guzeloy, 2014) and half of them went so far as to describe it as a moderate or even severe social handicap. However, in the present study, the percentage of affected women who sought medical attention was not included as in some cases, a few years pass before the patient brings the problem to the attention of her physician.

The tendency of affected women, especially in traditional Cultures as ours, to hide the problem raises the question of whether it is up to the family physician to broach the subject. Gynecologic consultations are the most frequent in the Saudi system, perhaps because women here have more direct access to female gynecologists, with no need for a referral from the family physician, but not to urologists. Experience has shown that even direct questioning of women about UI arouses very little response (Pakgohar, 2014). Specific attention, especially in high risk populations, needs to be addressed to the problem and new methods sought to highlight it. A good rapport between patient and physician, and patient trust in the physician may encourage more women to discuss the problem.

## 5. Conclusions

Urinary incontinence is common and often disturbing middle aged Saudi women, our study found that the prevalence was almosr 34%. It adversely impaired their quality of life (QOL). Women`s age and parity were significantly related to impaired QOL.
